# A multicomponent family support intervention in intensive care units: study protocol for a multicenter cluster-randomized trial (FICUS Trial)

**DOI:** 10.1186/s13063-022-06454-y

**Published:** 2022-06-27

**Authors:** Rahel Naef, Miodrag Filipovic, Marie-Madlen Jeitziner, Stefanie von Felten, Judith Safford, Marco Riguzzi, Michael Rufer

**Affiliations:** 1 Institute for Implementation Science in Health Care, Faculty of Medicine, University of Zurich, Universitätsstrasse 84, 8006 Zurich, Switzerland; 2 Centre of Clinical Nursing Science, University Hospital Zurich, Rämistrasse 100, 8091 Zurich, Switzerland; 3 Surgical Intensive Care Unit, Division of Anesthesiology, Intensive Care, Rescue and Pain Medicine, Cantonal Hospital of St. Gallen, Rorschacher Strasse 95, 9007 St. Gallen, Switzerland; 4 Department of Intensive Care Medicine, University Hospital Bern, Inselspital, University of Bern, Freiburgstrasse 16, CH10, Bern, Switzerland; 5 Department of Biostatistics, Epidemiology, Biostatistics, and Prevention Institute, Faculty of Medicine, University of Zurich, Hirschengraben 84, 8001 Zurich, Switzerland; 6 Bern, Switzerland; 7 Department of Psychiatry, Psychotherapy, and Psychosomatics, Psychiatric University Hospital Zurich, University of Zurich, Zurich, Switzerland; 8 Center for Psychiatry and Psychotherapy, Clinic Zugersee, Triaplus AG, Widenstrasse 55, 6317 Oberwil-Zug, Switzerland

**Keywords:** Intensive care (MeSH), Family (MeSH), Family nursing (MeSH), Anxiety (MeSH), Depression (MeSH), Post-traumatic stress disorder (MeSH), Postintensive care syndrome – family (non-MeSH), Satisfaction with care (non-MeSH), Cluster-randomized controlled trial (non-MeSH)

## Abstract

**Background:**

Family members of critically ill patients face considerable uncertainty and distress during their close others’ intensive care unit (ICU) stay. About 20–60% of family members experience adverse mental health outcomes post-ICU, such as symptoms of anxiety, depression, and posttraumatic stress. Guidelines recommend structured family inclusion, communication, and support, but the existing evidence base around protocolized family support interventions is modest and requires substantiation.

**Methods:**

To test the clinical effectiveness and explore the implementation of a multicomponent, nurse-led family support intervention in ICUs, we will undertake a parallel, cluster-randomized, controlled, multicenter superiority hybrid-type 1 trial. It will include eight clusters (ICUs) per study arm, with a projected total sample size of 896 family members of adult, critically ill patients treated in the German-speaking part of Switzerland. The trial targets family members of critically ill patients with an expected ICU stay of 48 h or longer. Families in the intervention arm will receive a family support intervention in addition to usual care. The intervention consists of specialist nurse support that is mapped to the patient pathway with follow-up care and includes psycho-educational and relationship-focused family interventions, and structured, interprofessional communication, and shared decision-making with families. Families in the control arm will receive usual care. The primary study endpoint is quality of family care, operationalized as family members’ satisfaction with ICU care at discharge. Secondary endpoints include quality of communication and nurse support, family management of critical illness (functioning, resilience), and family members’ mental health (well-being, psychological distress) measured at admission, discharge, and after 3, 6, and 12 months. Data of all participants, regardless of protocol adherence, will be analyzed using linear mixed-effects models, with the individual participant as the unit of inference.

**Discussion:**

This trial will examine the effectiveness of the family support intervention and generate knowledge of its implementability. Both types of evidence are necessary to determine whether the intervention works as intended in clinical practice and could be scaled up to other ICUs. The study findings will make a significant contribution to the current body of knowledge on effective ICU care that promotes family participation and well-being.

**Trial registration:**

ClinicalTrials.gov NCT05280691. Prospectively registered on 20 February 2022.

**Supplementary Information:**

The online version contains supplementary material available at 10.1186/s13063-022-06454-y.

## Administrative information

**Table:** 

Title (1)	A multicomponent family support intervention in intensive care units: study protocol for a multicenter cluster-randomized trial (FICUS Trial)
Trial registration (2a and 2b).	ClinicalTrials.gov, NCT05280691, and the Swiss National Clinical Trials Portal (SNCTP), SNCTP000004842
Protocol version (3)	Version 1.0, 25. October 2021
Funding (4)	The study is funded by the Swiss National Science Fund (SNSF, grant no. 33IC30_198778/1).
Author details (5a)	**Rahel Naef:** Institute for Implementation Science in Health Care, Faculty of Medicine, University of Zurich and Center of Clinical Nursing Science, University Hospital Zurich, Universitätsstrasse 84, CH-8006 Zurich, Switzerland. **Miodrag Filipovic:** Surgical Intensive Care Unit, Rescue and Pain Medicine, Intensive Care, Division of Anesthesiology, Cantonal Hospital of St. Gallen, Rorschacher Strasse 95, CH-9007 St. Gallen, Switzerland. **Marie-Madlen Jeitziner:** Department of Intensive Care Medicine, University Hospital Bern, Inselspital, University of Bern, Freiburgstrasse 10, 3010 Bern, Switzerland. **Stefanie von Felten:** Department of Biostatistics, Epidemiology, Biostatistics, and Prevention Institute, Faculty of Medicine, University of Zurich, Hirschengraben 84, 8001 Zurich, Switzerland. **Judith Safford:** Patient representative, no affiliation **Marco Riguzzi:** Institute for Implementation Science in Health Care, Faculty of Medicine, University of Zurich and Center of Clinical Nursing Science, University Hospital Zurich, Universitätsstrasse 84, 8006 Zurich, Switzerland. **Michael Rufer:** Department of Psychiatry, Psychotherapy, and Psychosomatics, Psychiatric University Hospital Zurich, University of Zurich and Center for Psychiatry and Psychotherapy, Psychiatric Hospital Zugersee, Triaplus AG, Widenstrasse 55, 6317 Oberwil-Zug, Switzerland.
Name and contact information of the trial sponsor (5b)	Rahel Naef, PhD, RN Institute for Implementation Science in Health Care, Faculty of Medicine, University of Zurich and Center of Clinical Nursing Science, University Hospital Zurich, Universitätsstrasse 84, CH-8006 Zurich, Switzerland. E-Mail: rahel.naef@uzh.ch Phone: +41 44 634 37 49
Role of sponsor (5c)	The sponsor-investigator acts as the coordinating investigator of the trial and has a primary role regarding study design; collection, management, analysis, and interpretation of the data; and writing of the report. She has ultimate authority over these activities. The funder (Swiss National Science Fund) has no role in the development of the study design; collection, analysis, or interpretation of the data; writing of the manuscript; or decision to submit the manuscript for publication.

## Introduction

Family members are important to the well-being and recovery of critically ill persons, yet they are themselves profoundly affected by the critical illness [1, 2]. During a close other’s treatment in an intensive care unit (ICU), families experience high levels of stress and uncertainty [3], which often negatively affects their coping ability and mental health [4–6]. Research reports that 20–60% of family members experience subsequent adverse mental health outcomes, such as symptoms of anxiety, depression, posttraumatic stress, and complicated grief [7, 8], also known as post-intensive care syndrome – family (PICS-F) [9–11]. With the COVID-19 pandemic, incidences of PICS-F are likely to be even higher [12, 13].

The impact of critical illness on family members’ health and well-being and the need to increase family access, inclusion, and support are gaining recognition [10, 14–16], even more so since the COVID-19 pandemic [13, 17–19]. A move towards structured inclusion, communication, and support of family is recommended [20]. While an abundance of research exists about how to better address families’ needs during critical illness, empirical knowledge about the effectiveness and successful implementation of family interventions and models of care is only modest.

### State of evidence on family interventions in ICU

Prior research has identified a promising effect of interventions seeking to increase family inclusion, communication, and support in ICU, such as participation in patient care or rounds, information/education, structured communication, or ICU diary, on quality of family care and family health outcomes [21–26]. While there is a general trend towards improved communication, decision-making, and family satisfaction and reports of reduced symptoms of anxiety, depression, and post-traumatic stress following such family interventions, effects were mostly found to be statistically non-significant with unclear clinical significance. The effects on length of ICU stay remain controversial [21, 22, 27], and most studies have used non-controlled designs [24, 25].

Studies that investigated specific, multicomponent, bundled family interventions, which included specific family support roles combined with facilitated communication, have demonstrated an increase in satisfaction with care and quality of communication [28–32], and have established feasibility and acceptability of family liaison and navigator roles [28, 31, 33, 34], most recently in the context of the COVID-19 pandemic [35–37]. However, there is little evidence of the effectiveness of such interventions to reduce symptoms of anxiety, depression, or posttraumatic stress [30, 32, 34, 38, 39]. Only three randomized trials on family support interventions in ICU have been conducted to date. Curtis and colleagues [38], testing a communication facilitator intervention for family members of incapacitated patients involved in surrogate decision-making, found a decrease in depressive symptoms but not in anxiety 6 months post-ICU, and there was no statistically significant difference after 3 months. White and colleagues [30], who investigated a multicomponent, nurse-delivered family support intervention (FOUR SUPPORT) for the same target group, did not identify a clear trend. Just recently, Kentish-Barnes and colleagues [39], who implemented a physician-driven, nurse-aided support strategy for family members of patients dying in ICU following a decision to withdraw or withhold life support, found a significant reduction in the proportion of family members with prolonged grief symptoms and significantly lower grief scores in the intervention group.

### Trial rationale

There is a clear clinical need for increased family communication and support during critical illness to improve evidence-based ICU care delivery [40, 41]. However, there is a lack of evidence-based family support programs. Our group has developed and pilot-feasibility tested a nurse-delivered family systems intervention program in a general ICU population using a mixed-method design. We were unable to demonstrate a favorable impact on post-ICU psychological distress, possibly due to the uncontrolled before-and-after comparison [32], but found statistically significant improved satisfaction levels. The qualitative evaluation showed self-perceived benefits for family management of critical illness and its aftermath when the intervention is initiated shortly after ICU admission, responsive to families’ unique needs, and perceived to be delivered with high proficiency [32]. The pre-existing evidence base around multicomponent family support interventions is modest and requires further substantiation, particularly using randomized controlled designs [24–26]. Hence, the here proposed trial seeks to generate high-quality evidence on the intervention effectiveness of such multicomponent, bundled family support interventions in ICU while also generating knowledge around implementation processes and outcomes.

## Objectives

**Fig. 1:**
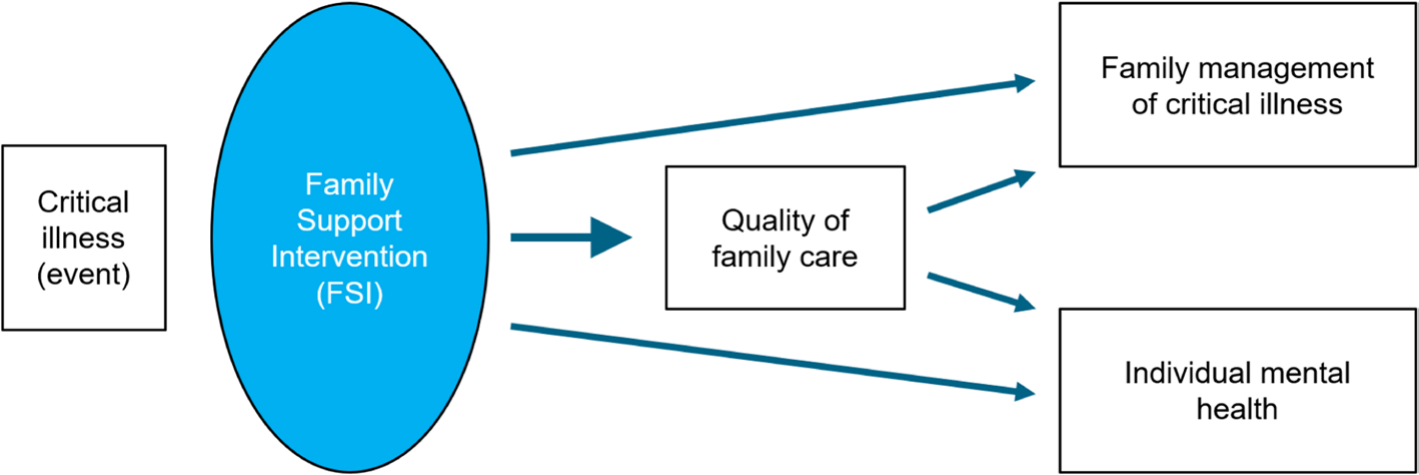
Intervention mechanism

The primary aim of the Family in Intensive Care UnitS (FICUS) trial is to test the effectiveness of a multicomponent, nurse-led family support intervention (FSI), on the quality of family care, family management of critical illness, and mental health of individual index family members of critically ill patients compared with usual ICU care (Fig. 1). We hypothesize that the FSI will (1) increase the quality of family care, i.e., satisfaction with ICU care (primary endpoint); (2) increase the quality of communication and nurse support as reported by family members after patient discharge/death; (3) improve family management of critical illness and individual well-being; and (4) decrease symptoms of psychological distress post-ICU. The secondary aim is to examine the implementation processes, that is, identify implementation barriers/enablers in the real-world context in which the study intervention is implemented to discern determinants and strategies of implementation success.

## Design and methods

The FICUS trial is designed as a parallel, cluster-randomized, controlled, multicenter superiority hybrid type 1 trial with an equal number of clusters per study arm and a primary endpoint of quality of family care in ICU assessed after patient ICU discharge or death. We employ an effectiveness-implementation hybrid design type 1 [42], in which, alongside clinical effectiveness, contextual determinants (barriers/facilitators), together with implementation processes and outcomes are explored. This protocol publication follows the SPIRIT guidance [43] (see the SPIRIT Checklist as an additional file to this manuscript).

### Patient and public involvement

A patient and family advisory group (PFAG) with five members (one patient, three family members, one patient representative) ensures that the trial is relevant and meaningful to critically ill patients and their family members, contributes to the design of the study processes, and guarantees that participation is feasible and acceptable to family members [44–46]. An ongoing partnership has been formed between the research team and the PFAG, with one member acting as a Patient and Public Involvement (PPI) liaison. This person co-designed the involvement strategy, co-leads consultation and communication, and acts as a liaison between the research team and the PFAG. Furthermore, PFAG members give advice, contribute their expertise to the trial design, and are actively involved in trial implementation.

To date, users have advised on the trial conception, namely the recruitment strategy, study outcomes, data collection processes, and the study intervention. They have provided feedback on the study protocol before submission to the funding body, and co-written user involvement strategy and lay abstracts. Future involvement during the preparatory phase includes the preparation of study information sheets, training of data assessors and interventionists, and communication recommendations for both the general public and for the recruitment of trial participants. The PFAG will continue to be engaged in the recruitment, data collection (i.e., retention procedures, ongoing reflection on study course), data analysis (i.e., interpretation of results, critical review), and dissemination (i.e., planning, dissemination activities, lay summaries) phases.

### Participants and setting

#### Study setting

The FICUS trial will take place in ICUs in the German-speaking part of Switzerland.

#### Cluster-level eligibility criteria

Study ICUs need to be able to offer the highest level of patient care and treat patients who are hemodynamically unstable, require ventilation with multiple-organ failure, and need multidisciplinary intervention [47]. They may offer different or combined specialty care, including surgical, trauma, medical, cardiac, or neurological care. ICUs certified by the Swiss Society for Intensive Medicine (SGI) to run at least eight beds are eligible. ICUs with less than 300 admissions per year of patients with an ICU stay of 48 h or longer are excluded, as are ICUs with a protocolized, interprofessional family support program.

#### Participant-level eligibility criteria

Participants are adult family members (≥ 18 years of age) of critically ill persons who receive treatment in an eligible ICU for at least 48 h. Critically ill persons are defined as those with an expected length of stay in ICU of ≥ 48 h, combined with a life-threatening condition with a high risk of death or long-lasting functional impairment, or a high risk of prolonged mechanical ventilation (> 24 h) as appraised by the intaking clinician. Family members are defined as close others from the patient’s perspective, as noted in clinical records or advanced directives, or as legally defined surrogate decision-makers. Legal or blood kinship is not a requirement. They have to be a primary support person of the critically ill person, able to complete the baseline data collection and family-reported questionnaires in German within the required time frame and sign a written informed consent form. Family members of patients with refused general consent will not be eligible to take part. Family members with prior inclusion in the FICUS trial in another ICU, with cognitive inability to understand the study or inability to complete the questionnaire as appraised by clinicians or study recruitment staff, will be excluded. Inability to complete the baseline data collection within the required time frame after admission/study enrollment will lead to exclusion [48].

#### Informed consent processes

The investigator or his/her delegates will be responsible for obtaining written informed consent. They will explain to each potential family member participant the nature of the study, its purpose, the procedures involved, the expected duration, the potential risks and benefits, and any discomfort it may entail. Each participant will be informed that the participation in the study is voluntary, that s/he may withdraw from the study at any time, and that withdrawal of consent will not affect his/her or their close other’s care. Family members will also be asked to state in an additional consent form whether their data obtained in the context of this trial may be used for secondary data analyses.

To extract clinical patient data, consent will be obtained from patients. If the patient has pre-signed a general consent form for the use of routine clinical data for research purposes, no additional consent form is required. If not, and in case the patient lacks cognitive ability to do so upon ICU admission, the participating family member will provide his/her surrogate written informed consent based on the presumed will of the critically ill patient. As soon as the critically ill patient regains his/her ability of judgment, his/her written informed consent will be obtained.

### Study intervention

The family support intervention (FSI), which has been developed and pilot-tested by the coordinating investigator [32, 33], has been slightly adapted in consultation with five users, seven ICU nurses, and three physicians to increase the implementability across the different study ICUs while maintaining its core components [49]. The aims of the FSI are (1) to increase the quality of interaction and communication between families and the ICU team and (2) to improve the ICU team’s capacity to provide the necessary care and support to families. The intervention also seeks (1) to strengthen family illness management capacity and well-being and (2) to alleviate the impact of the critical illness and/or loss on family members’ mental health.

#### Explanation of choice of comparator

The FSI will be introduced to the study ICUs of the intervention arm in addition to usual care, which is the control condition.

#### Usual care

Usual care is defined as a non-protocolized approach to family care and services offered by nurses, physicians, teams, or other health professionals that are an established part of the ICU’s routine care delivery before the trial start. These include granting access (visitation), interacting with family members and informing about the patient’s condition and treatment (written and oral information), communicating with family members as surrogate decision-makers (communication structure), supporting families (support structures), and making referrals (auxiliary services). ICU staff in control units will be allowed to act upon patient/family needs. However, the introduction of a new protocolized family support intervention, a family nursing or other family support role, or a structured family support pathway is not permitted.

#### Intervention description

**Fig. 2:**
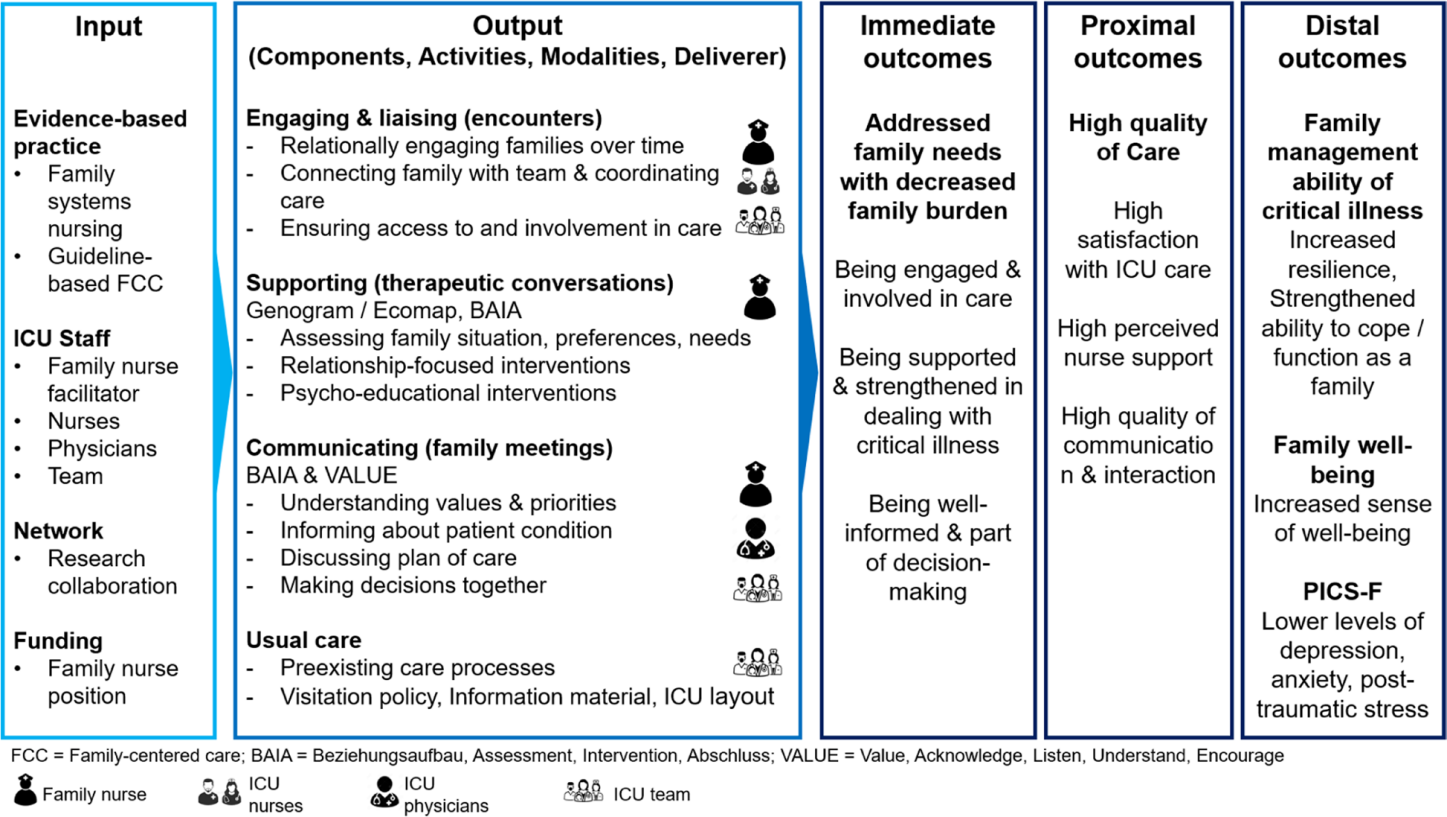
FSI program logic model

The FSI is grounded in a family-systems nursing approach [50–53]. It proposes that critical illness affects families’ affective, cognitive, and behavioral functioning, requiring therapeutic relational nurse-family engagement including relationship-focused and psycho-educational interventions to support family management of illness and to alleviate their suffering. The development was based on evidence around systemic family interventions for chronic illness [54–57], which suggests that the use of a combination of psycho-educational and relationship-focused interventions is most effective in strengthening family and individual health and well-being. The FSI also builds on guideline-based recommendations around ICU care to families that include the use of a specific family consultation role and of structured, interprofessional communication with families to ensure high quality of family care [15] (Fig. 2).

**Fig. 3:**
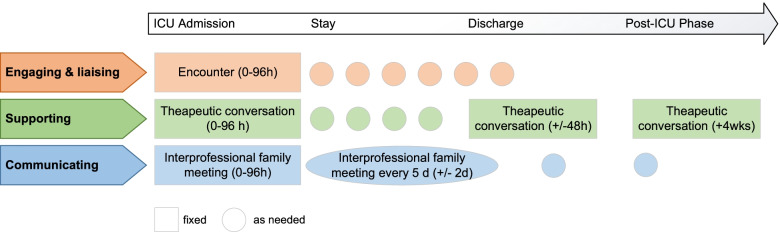
Family care pathway

The FSI consists of a new family nursing role that delivers three interacting, closely intertwined intervention components that are mapped to a family care pathway (Fig. 3):

- *Engaging and liaising (family encounters)*: This intervention component involves relationship-building with families, connecting and coordinating family care activities as well as transition and follow-up care for family members and surviving patients.
- *Supporting (therapeutic family conversations)*: This component is based on a relational family systems nursing approach and includes assessing family structure, processes, and resources and supporting families through relationship-focused and psycho-educational interventions at the family systems level.
- *Communicating (interprofessional family meetings)*: This component focuses on structured, interprofessional communication, and shared decision-making with families.

The FSI will be delivered by two to three designated family nurses per ICU in close collaboration with the ICU team. Interventionists will be registered nurses with a certification in ICU nursing or equivalent and training in family systems nursing. They hold a Master of Science in Nursing degree or work under the supervision of an advanced practice nurse. Advanced competencies in family nursing [58] are necessary for the following three reasons: First, families are in a vulnerable situation and/or crisis, which requires specific knowledge and expertise around family processes and illness management. Second, the relational, systemic family interventions require specific knowledge and skills beyond the general competencies of ICU nurses. Third, interventionists will assume a combined role encompassing clinical practice with families, consultation, collaboration, and leadership.

#### Criteria for discontinuing/modifying interventions

As this is a cluster-randomized trial (RCT), there will be no individual-level assignment that could be discontinued. However, the intervention will be discontinued if a family member withdraws the consent.

#### Strategies to improve adherence to interventions

The intervention is standardized in terms of components, intervention content, and a minimum dose along the clinical patient pathway (see Fig. 3) [59]. The frequency of intervention contacts and the dose of each intervention component can be increased and tailored according to the patients’ and/or families’ preferences and needs. Individual patient or family adherence to a particular health behavior is not required. Fidelity to intervention protocol is ensured at the individual and cluster levels. Adherence will be promoted by a 5-day interventionist training, monthly supervision and case conferences with all study interventionists, and site visits for quality assurance purposes.

#### Relevant concomitant care permitted/prohibited

The intervention components will be delivered within predefined time windows along the patient pathway, that is, within 96 h after admission, 48 h before or after discharge, and within the first 4 weeks after ICU discharge. A higher intervention dose and frequency of each intervention component are permitted to tailor to patient illness course, length of ICU stay, and family needs and preferences. Any interaction between ICU shift or primary nurses, ICU, or other treating physicians and other health or social care professionals is explicitly permitted. There are no medical interventions that are prohibited during the study.

#### Implementation strategy

The FSI will be implemented using a combination of strategies that showed promise in the pilot study [33]. These implementation strategies include leadership endorsement, nurse and physician champions, team education, and external/internal implementation support practitioners [60]. They will be adapted and tailored to the local context to address potential barriers, which will be identified prior to the enrollment start [61, 62].

### Outcomes

#### Primary outcome

The primary outcome is the quality of family care in the ICU, operationalized as family satisfaction with ICU care, which is an established core indicator of the quality of family care [63–65]. It will be assessed at discharge from ICU by the Family Satisfaction in ICU Questionnaire (FS-ICU-24R) [66, 67].

#### Secondary outcomes

Quality of family care is further operationalized as the quality of communication and nurse support (see Table 1 for measures). As the intervention also targets families’ ability to cope with critical illness, family management of critical illness was chosen as a further secondary outcome. Individual family members’ mental health will be operationalized to cover the spectrum from well-being to psychological distress. Family management and mental health indicators are more distal than the quality of family care but highly relevant outcomes of interventions that address families’ needs for support and promote family capacity and health [68, 69] (Fig. 1). They will be obtained at baseline, discharge, and 3, 6, and 12 months thereafter.

**Table 1:** Individual-level primary and secondary outcomes

Domain/construct	Measure^**a**^	Range	Cronbach’s ***α***^**b**^	T0	T1	T2	T3	T4
**Quality of family care**								
Satisfaction with care (primary outcome)	Family satisfaction with ICU care (FS-ICU-24R)	0–100	> .85		X			
Quality of communication	Questionnaire on Quality of Physician-Patient Interaction (QQPPI-14)	1–5	.95		X			
Support from nurses	Family Perceived Support Questionnaire (ICE-FPSQ-14)	14–70	> 0.87		X			
**Family management**								
Family functioning	Family Assessment Device - General Functioning Scale (FAD-GF-12)	1–4	.87	X	X	X	X	X
Family resilience	Brief Resilience Scale (BRS-6)^c^	1–5	.85	X	X	X	X	X
**Mental health**								
Subjective well-being	Satisfaction with Life Scale (SWLS-5)	5–35	.89–.92	X	X	X	X	X
	WHO-5 Well-Being Index (WHO-5)	0–100	.92	X	X	X	X	X
	Adapted VAS on Quality of Life (QoL-VAS)	0–100	n/a	X	X	X	X	X
Psychological distress	Distress Thermometer (DT)	0–10	n/a	X	X	X	X	X
	Impact of Events Scale-6 (IES-6)	0–4	.80	X	X	X	X	X
	Hospital Anxiety and Depression Scale (HADS-14)	0–21	> .80	X	X	X	X	X

^a^Use of German versions of measures

^b^References for Cronbach’s alpha are listed in the text

^c^Adapted for family

#### Cluster-level data

Data will also be obtained at the cluster level, that is, from participating ICUs, and include data on ICU characteristics and family care processes and policies.

#### Process data

Process data will be obtained in the intervention arm, namely information on implementation activities and costs (cluster level) and on intervention delivery (individual level).

#### Accompanying studies

To address the second study aim, a three-phased implementation study will be undertaken among the clusters of the intervention arm. First, a context assessment will be conducted to identify barriers to the implementation of FSI in the specific ICU. Next, a tailored implementation plan will be developed, and an adaptive process put in place. Then, the implementation process and outcomes will be evaluated using a mixed-method approach with a case-study format.

An economic evaluation will be undertaken to assess the cost associated with intervention delivery and implementation of the FSI. Working hours per intervention (FSI) will be analyzed in relation to family members’ mental health and their absenteeism/presentism at work.

### Cluster and participant timelines

Eligible clusters, that is, ICUs that have signed a study agreement, will be enrolled and randomly assigned to the intervention or control arm after cluster-level baseline data collection has been completed. Cluster data will be obtained at baseline, then yearly, and finally after the conclusion of the discharge data collection (first follow-up) of the last participant.

Participant eligibility screening and enrollment will be conducted at the individual level using a consecutive sampling strategy. The participant timeline is displayed in Table 2.

**Table 2:** Participant timeline

Task/study period	Admission to ICU	Screening	Informaiton	ICF	Baseline assessment (T0)	FU 1 to discharge from ICU (T1)	EOT and FU 2–3 months (T2)	FU 3–6 months (T3)	FU 4–12 months (T4) = EOS
Time point	0–24 h	0–48 h	0–72 h	0–96 h	0–96 h	Y (− 24 h/+ 14 days)	Y + 90 days (± 14 days)	Y + 180 days (± 14 days)	Y + 365 days (± 14 days)
**Enrollment**									
Eligibility screen of patient	X								
Identification of eligible family member		X							
Information of family member			X						
Informed consent				X					
**Interventions**									
Intervention arm: family support intervention including intervention log					X	X	X		
Usual care	X	X	X	X	X	X			
**Assessments**									
Patient demographics, health and functional status, care utilization^a^				X	X	X	X	X	X
Family member demographics, self-perceived health, care utilization					X	X	X	X	X
Satisfaction with care (FS-ICU-24R) (primary endpoint)						X			
Quality of communication (QQPPI-14)						X			
Nurse support (ICE-FPSQ-14)						X			
Family functioning (FAD-GF-12)					X	X	X	X	X
Family resilience (BRS-6)					X	X	X	X	X
Distress Thermometer (DT)					X	X	X	X	X
Depression and anxiety (HADS-14)					X	X	X	X	X
Posttraumatic stress (IES-6)					X	X	X	X	X
Life satisfaction (SWLS-5)					X	X	X	X	X
Well-being (WHO-5, QoL-VAS)					X	X	X	X	X

*ICF* informed consent form, *FU* follow-up, *EOT* end of treatment, *EOS* end of study, *Tx* assessment time point

^a^T0–T1 from clinical record, T2–T4 family member reported

### Sample size

Assuming a difference in FS-ICU-24 total score between the intervention and control groups of 5.5 and a within-group standard deviation of the FS-ICU-24 total score of 16.3, as observed in Naef and colleagues [32], we would need a total of 278 subjects in a standard RCT (with *n*_RCT_ = 139 per arm) with 1:1 randomization to have a power of 0.8 at a significance level of 0.05. However, due to the *design effect* [70], the number of participants required for our cluster-randomized trial is bigger than the sample size of a standard RCT. We assumed an intra-cluster correlation coefficient (ICC) of 0.03 and a coefficient of variation in cluster size of 0.2.

Since the number of clusters is more limiting for this study than cluster size, we used the approach described by Hemming and colleagues [71] to derive the minimum number of clusters required for each condition in our trial. Assuming an unlimited cluster size, the minimum number is simply *n*_RCT_ × ICC = 5 clusters. Increasing the number of clusters to eight (three more than the minimum), the cluster size could be reduced to $\frac{n_{\mathrm{RCT}}}{3}$=47.

We then conducted a series of precise power calculations, using the R packages *clusterPower* [72] and *sse* [73], considering a range of clusters per condition (3,…,20), mean cluster sizes (10–300), and ICCs (0.01–0.1). With an average cluster size of 50 evaluable patients and an ICC of 0.03, 8 clusters need to be randomized to each condition to achieve a power of 0.8. To account for a drop-out rate of 10% of patients within clusters (but no drop-out of whole clusters), an average number of 56 patients should be recruited per cluster.

### Recruitment

Eligible ICUs in German-speaking Switzerland were identified based on the list of Swiss Society for Intensive Medicine-certified ICUs. The coordinating investigator contacted eligible ICUs to invite participation and enroll them in the study.

Eligible family members will be recruited within 96 h after patient admission to ICU. Designated ICU nurses or physicians will perform daily eligibility screening of newly admitted patients and potential family participants. They will obtain contact details of at least one family member and provide initial information to family members and hand out the study flyer and information. Study flyers will also be displayed in the waiting area and included in ICU information packages routinely handed to family members. Written study information will also be available on websites.

Next, the clinician or study coordinator will contact eligible family members in person when they visit their critically ill close other or via phone, explain the study, invite participation, and enroll participants. Family members will receive time to decide for or against participation in accordance with ethical guidelines. Participants will not receive payment or any other form of compensation. A thank you letter will be written to them upon study completion.

### Assignment of interventions

Clusters will be assigned 1:1 to the intervention and the control arms. To reduce a potential imbalance of study groups at baseline, restricted randomization will be used [74, 75].

#### Assignment

The assignment of the participating ICUs will be generated after their recruitment and baseline cluster data collection using minimization at a central location. Variables used in the minimization procedure are the degree of specialization (specialized vs. general ICU) and the hospital. The first cluster will be assigned completely at random. To avoid subsequent assignments being deterministic, a random component of 10% will be introduced.

#### Concealment mechanism

The assignment will be generated by the trial statistician and only be revealed to the investigators after cluster-level baseline data collection has been completed.

#### Implementation

Clusters will be identified and enrolled by the coordinating investigator. The cluster assignment will be generated by the trial statistician, using the R package *Minirand* [76]. The coordinating investigator will then communicate the assignment to the local investigators.

#### Blinding

This is a non-blinded study. Blinding of study participants and clinical and research staff will not be possible due to the nature of the study intervention and cluster design.

### Data collection and management

#### Assessment and collection of cluster-level data

Data at the cluster level will be collected by a paper case report form (pCRF) at baseline before cluster assignment to a study arm, every 12 months, and after the last participant in each cluster has completed the first follow-up (discharge).

##### ICU characteristics

ICU characteristics, such as the number of beds, admissions per year, percentage of high risk and mechanically ventilated patients, length of stay, mortality rate, will be obtained from the most recent Minimal Data Set (MDSi), which is routinely collected using a standardized data capture system provided by the Swiss Society of Intensive Medicine.

##### Quality of family care

To describe usual care processes offered to family members in study ICUs prior to and during the study, a brief form and two self-assessment instruments will be completed by a team of ICU clinicians. These instruments capture usual care processes and family care policies pre-intervention and allow to distinguish between usual care and the FSI post-intervention at the cluster level.

*Self-Assessment Tool for Family-Centered Care in ICU* [77, 78], which is based on the Society of Critical Care Medicine’s guideline for family-centered care [15]. The tool assesses on a 4-point Likert scale the degree to which recommended family engagement practices are currently delivered, such as family presence, support, communication, consultation, and family-friendly layout.

*Patient- and Family-Centered Care Organizational Self-Assessment Tool* [79] assesses the degree of family engagement at the hospital level, using eleven domains, such as leadership, quality improvement, personnel, mission & visions, and charting and documentation.

##### Implementation log

Implementation strategies used to introduce and maintain the fidelity of the intervention will be recorded by an internal implementation facilitator or local investigator in a structured format. Cost indicators include (but are not necessarily limited to) implementation of structures and processes, acquisition of staff to deliver the intervention, and intervention-specific training.

#### Assessment and collection of individual-level data

All participants who were assessed for study eligibility, irrespective of whether they were enrolled, considered non-eligible, or were eligible but not enrolled in the study, have to be recorded in a screening log. Investigators will document each study participant in an enrollment log and generate an individual participant schedule. Individual participant-level data will be collected by local study coordinators with an electronic case report form (eCRF), after participant enrollment at admission (baseline), discharge, and 3, 6, and 12 months thereafter via online or paper/pencil survey from family members and via direct data entry from clinical records. To access the online survey, a personalized link will be generated for each participant and assessment time point, which will be sent to participants via email, a text message, or other predetermined means. Participants can opt to answer the survey on a printed paper and pencil version of the eCRF, which will be sent to them with a stamped return envelope. Data collection at ICU admission and discharge will involve either phone or face-to-face interactions to facilitate the timely completion of the survey during this particularly vulnerable phase of a critical illness. During the post-ICU phase, emails or letters will be sent to family members to inform them about the upcoming follow-up data collection. If the survey is not completed within 2 weeks, study coordinators will be followed up by phone every week for 2 weeks. Follow-up calls for data collection reasons will be recorded in the participant list.

##### Outcome measures obtained via survey

The following family-reported outcome measures will be used in their German versions as part of the family member survey (see Table 1):

*Family satisfaction in ICU (FS-ICU-24R)*: The FS-ICU-24-R yields a transformed mean score for overall satisfaction, satisfaction with care (16 items), and satisfaction with involvement in decision-making (ten items). The test-retest reliability of this assessment was high in the original pilot *r* = .85 [65], and the instrument is sensitive to change in several intervention studies. The German version of the FS-ICU-24R has demonstrated no floor or ceiling effects [66, 67].

*Questionnaire on the Quality of Physician-Patient Interaction (QQPPI)*: The QQPPI assesses relationship-building, information exchange, and shared decision-making (14 items) and has been appraised as one of the most psychometrically sound measures of health professional-patient interaction [80] with a test-retest reliability of .59 [81].

*Family Perceived Support Questionnaire (ICE-FPSQ)*: The ICE-FPSQ-14 measures families’ perception of support provided by nurses on two subscales: emotional support (nine items) and cognitive support (five items) [82, 83]. It has been translated into German employing a systematic procedure [84] and is currently under psychometric validation by the research group.

*Family Assessment Device - General Functioning Scale (FAD-GF)*: The FAD-GF-12 is used to assess the overall functioning of the family system [85–87] and has demonstrated good test-retest reliability (*r* = .60 after 12 weeks) among patients with PTSD as well as responsiveness in intervention studies [88].

*Brief Resilience Scale (BRS)*: The BRS-6 is conceived to measure the essence of resilience as the ability to bounce back from stress [89], and evidence from intervention studies suggests that the scale is sensitive to change [90, 91]. Its items will be reformulated from “I” to “we” statements to assess family rather than individual ability.

*Satisfaction with Life Scale (SWLS-5)*: Developed to measure the global dimension of subjective well-being [92], the SWLS-5 can detect changes over time as well as responses to treatment [93]. The sum of the scores (range 5–35) can be transformed to an ordinal scale with seven levels ranging from *extremely dissatisfied* to *extremely satisfied* (with 20 indicating neutrality).

*WHO-5 Well-being Index (WHO-5)*: The WHO-5 was developed to measure subjective psychological well-being [94]. It is widely used in European surveys [95, 96] and can detect clinically relevant changes while a score ≤ 50 (on a 0–100 scale) is indicative of depression.

*Adapted VAS on Quality of Life (QoL-VAS)*: An adapted version of a visual analog scale (VAS) similar to that used in the EuroQol EQ-5D Questionnaire [97] will measure the self-perceived general quality of life (QoL) rather than health-related QoL.

*Distress Thermometer (DT)*: The Distress Thermometer is originally and still primarily used among cancer patients [98–102], yet it is a generic visual analog scale (VAS) [103]. A common cutoff of 4 indicates potential distress [98, 104], and the thermometer can detect changes over time [105–107].

*Impact of Events Scale-6 (IES-6)*: The IES-R is a widely used instrument to assess psychological distress in ICU family members [3, 108], which measures the presence and severity of symptoms associated with a traumatic event during the past week. The brief version (IES-6) includes two items from each of the three subscales of the IES-R-22 version (intrusion, avoidance, hyperarousal) [109, 110], is highly correlated to the IES-R [111], and detects changes over time [110].

*Hospital Anxiety and Depression Scale (HADS)*: HADS-14 [9, 108, 112] is scored on two subscales (anxiety and depression) and has thresholds for mild/caseness for depression and anxiety, respectively [9, 108, 112]. The HADS has been shown to be an effective measure of psychological distress [113] and can detect changes over time [114, 115] as well as varying degrees of severity [115].

##### Further family member- and patient-related data

At baseline and first follow-up, information on the patients’ health condition and ICU treatment will be extracted from the clinical record. After that, family members will be asked to provide proxy information on patients’ care utilization and functional status. Family members will complete a demographic form together with information on their work situation, health status, and care utilization as part of the survey at each assessment time point (Table 3).

**Table 3:** Patient- and family member-related data

Time point of assessment	Patient					Family member				
	T0	T1	T2	T3	T4	T0	T1	T2	T3	T4
**Demographics**										
Age, gender, civil status (T2–T4 change), Education	X					X		X	X	X
Type of family member (T0), primary carer (yes/no)						X		X	X	X
Co-habiting with patient (yes/no), frequency of contact						X				
Travel time to hospital						X				
**Health status**										
Medical diagnosis (MDSi)	X	X								
Trauma (yes/no); if yes, AIS^a^	X									
Mechanical ventilation and circulary support (T0: yes/no, T1: # of days)	X	X								
SAPS-2^b^	X									
NEMS^c^, SOFA^d^	X	X								
Death (yes/no); if yes, date, cause, place^e^	X	X	X	X	X					
Organ donation (yes/no); if yes, organ, surrogate dm)	X									
Functional status (Katz ADL/Lawton IADL)^e^			X	X	X					
Prior/present psychiatric diagnosis (yes/no)						X				
Self-perceived health (VAS)						X	X	X	X	X
**Care utilization**										
Cause of admission	X									
Type of admission (expected, unexpected), admitted from	X									
Surgery (emergency, planned, none)	X									
Previous ICU stays (yes/no)	X									
Prior need for informal/ formal care (yes/no)^f^	X									
Length of ICU stay		X								
Discharge/transfer destination, planned vs. unexpected		X								
Need for informal/formal care (yes / no); if yes, change, hours/frequency, more/less than before ICU treatment)^e^			X	X	X					
Prior/current/new psychological/psychiatric treatment (yes / no)						X	X	X	X	X
Use of prescription drugs						X	X	X	X	X
Current treatment for a chronic condition (yes/no)						X				
Number of family physician visits/hospitalizations (if yes: related to critical illness yes/no/length of stay)							X	X	X	X
Previous ICU experience (as patient, as family of the patient)						X				
Family satisfaction with prior care experience (VAS)						X				
Family satisfaction with care on hospital wards (VAS)								X		
**Work situation**										
Level of employment, income						X				
Change in level of employment (yes / no); if yes: related to critical illness yes/no), new income							X	X	X	X
Sick leave/absence from work (if yes: # of (half-)days)						X	X	X	X	X
Presenteeism							X	X	X	X

^a^Abbreviated Injury Scale

^b^Sequential Organ Failure Assessment Score

^c^Nine equivalents of nursing manpower use score

^d^Simplified Acute Physiology Score

^e^Patient-related information at T2, T3, T4 obtained from family member

^f^Patient-related information at T0 obtained from family member

##### Intervention log

For each intervention contact, the following data will be recorded by interventionists in the eCRF: date, ICU or post-ICU day, duration in minutes, delivery mode (face-to-face, phone, online), number/type of family members and ICU staff present, type of intervention component (encounter, therapeutic conversation, interprofessional family meeting), nurse intervention activity (relational engaging, family assessment, psycho-educational intervention, relationship-focused interventions, liaison and coordination, interprofessional communication, shared decision-making, making referrals), and referrals to auxiliary services. Hence, all concomitant interventions or interventions exceeding usual care have to be recorded. Usual care itself will not be documented.

#### Retention and complete follow-up

To promote retention at the cluster level, the trial steering committee will hold quarterly study group meetings to discuss study progress, and provide quarterly communication via newsletters to study sites and staff. The trial manager and further members of the research team will interact regularly with the local study team and site.

To ensure retention of individual study participants, local study teams will make the utmost effort to obtain completed questionnaires at admission, discharge, and at the three post-ICU follow-ups at months 3, 6, and 12 [48]. As the study endpoints rely mainly on the completion of questionnaires by the participants, system-supported automatic notifications on the eCRF completion status will be implemented to ensure data completeness. Study coordinators will check the completeness of the paper-reported outcome measures and follow-up with participants if they identify missing values.

If participants withdraw consent, the reason for withdrawal will be assessed at the time point of withdrawal, and—if the participant agrees—data on the clinical status of the patient will be collected (change in health status/or death, time point of death). In this case, the intervention and the data collection will be discontinued, and no follow-up is planned. Participants who withdraw or are discontinued before the first follow-up (discharge) will be replaced to achieve the required sample size. The data of the last completed assessment time point will be included in the analysis (as per intention-to-treat analysis).

#### Data management

For data processing and management, the electronic data capture (EDC) system REDCap [116] will be used. A data monitoring plan specific to the study assessment schedule has been prepared by the coordinating investigator site’s clinical trial unit. Data monitoring will be initiated after the inclusion of ten study participants at each study center. Regular monitoring will be undertaken by the trial manager and/or external monitor. Observations and findings will be documented and made available to the coordinating investigator and local study site.

#### Confidentiality

Role- and user-based access control with personal login regulate permission to access the EDC system, which includes individual user rights for data entry, review, export, and reports. Appropriate coded identification is used to enter participant data into the database; no patient identifying information will be entered into the EDC system.

#### Data storage

The servers hosting the EDC system and study database are kept in an off-site restricted access locked server room. A copy of the study database will be stored securely by the Clinical Trial Unit at the coordinating investigator’s site for at least 10 years. Investigators maintain the essential documents and source data in the Trial Master File and Investigator Site Files and archive interim and final reports in electronic and hard copy format for at least 10 years.

### Statistical methods

A more detailed statistical analysis plan will be finalized before database closure. All analyses will be performed in R [117].

#### Statistical methods for primary and secondary outcomes

The FS-ICU-24-R total score at patient discharge from ICU will be analyzed by a linear mixed-effects model (LMM) with a random intercept per cluster to account for the non-independence of family members from the same cluster. Due to the small number of clusters, the main model will include the treatment (intervention vs. control) as the only explanatory variable in the main analysis, and the Satterthwaite approximation for the denominator degrees of freedom will be used, as recommended by Leyrat and colleagues [118]. The ICC will be estimated from this model based on the residual variance and between-cluster variance.

The following covariate-adjusted sensitivity analyses will be conducted to adjust the treatment effect estimate for potential confounding: At the cluster level, the specialization of the ICU (as used in the cluster randomization), overall ICU staffing, and a quality-of-care indicator will each be added separately to the main model described above. At the individual participant’s level, patient age, cause of admission, the SAPS-2 score of the patient (mortality risk), and the family member’s previous ICU experience will be added to the main model together (and potentially to the cluster-level adjusted models).

The secondary outcomes regarding the quality of care, which are only measured once at discharge from ICU, will be analyzed with an LMM as described above for the primary outcome. All other secondary outcomes, which are measured at baseline, at discharge from ICU, and at three other follow-up points in time, will be analyzed by an LMM with a random intercept per cluster and a random intercept per family member (nested within the cluster) to additionally account for the non-independence of repeated measurements from the same study participant. The serial autocorrelation of residuals will be modeled using a first-order autoregressive correlation structure. The models will include the treatment (intervention vs. control), the corresponding baseline measurement, the visit, and the visit-treatment interaction.

#### Methods for additional analyses

Subgroup analyses are planned regarding the primary outcome for the following baseline characteristics: specialization (specialized vs. general ICU), overall ICU staffing, and quality of care indicator (sum score) at the cluster level and patient age, gender, cause of admission (expected vs. unexpected), mortality risk (SPAS-2 score upon admission), type of relationship between patient and family member, family member prior ICU experience, family resilience (BRS-6), family functioning (FAD-FG-12), and anxiety and depression (HADS) at the participant level. A separate LMM will be fitted for each subgroup variable, adding the subgroup variable and the interaction between the subgroup variable and the treatment as explanatory variables to the main model. A significant interaction between a subgroup variable and the treatment would indicate a different treatment effect in the corresponding subgroups (or along a gradient for the continuous subgroup variables). Furthermore, patient survival status and ICU length of stay are two participant-level covariates that are of interest but are measured during or after the intervention and therefore do not qualify as baseline characteristics to adjust for in these subgroup models. However, for exploratory purposes, a model including these two variables together with the treatment and the three two-way interaction terms will be fitted, which will require cautious interpretation. Similarly, a model that includes the intervention dose will be fitted (with a dose of zero for the control group). No interim analyses are planned.

#### Methods in analysis to handle protocol non-adherence and missing data

The analysis will follow the intention-to-treat principle. Clusters and their participants will be analyzed given the condition to which they were assigned. Should a patient be transferred to another cluster, the family will be analyzed in the original cluster. This will be possible due to individual informed consent. The main analysis will be complete cases, but multilevel multiple imputation of missing data at the participant level [119] will be performed (potentially also to the covariate-adjusted models) to assess the sensitivity of the results with regard to missing outcomes. We do not expect any clusters to be missing as a whole but would exclude them from the analysis if present.

#### Plans to give access to the full protocol, participant-level data, and statistical code

The full protocol has been made available at ClinicalTrials.gov, NCT05280691. Participant-level data (after anonymization) and statistical code for data analysis will be made available upon reasonable request.

### Oversight and monitoring

A trial steering committee; that is, coordinating investigator, co-investigators, trial manager, data manager, statistician, implementation support practitioner, and patient representative, will oversee the trial implementation and conduct at the study sites. Data management and monitoring are provided by the coordinating investigator site’s clinical trial unit. The trial will follow national and international standards for good clinical practice and comply with regulatory and ethical requirements. Related or unrelated serious adverse events (SAEs) affecting the participants (family members) are collected and documented in source documents.

### Dissemination plans

The scientific output will be published and made available as widely as possible to support knowledge transfer, meta-analyses, and general reproduction efforts. All study centers and study participants will be provided with a lay summary of the study outcome (upon interest), and preliminary findings will be shared through poster presentations, scientific talks, and scientific publications on a national and international level at appropriate platform events typically attended by ICU staff, health care professionals, or the public.

## Discussion

The FICUS trial has the dual aim to establish the clinical effectiveness of the FSI, a nurse-delivered, interprofessional multicomponent intervention that is introduced into routine care delivery and to explore its implementation. In line with the MRC framework for developing and evaluating complex interventions [120], the current testing of intervention effectiveness and exploration of implementation builds on previous phases of the FSI development and feasibility-pilot testing [32, 33].

Given the state of research in the field of family intervention in the context of critical illness [22, 24–26, 121, 122], rigorous real-world evidence generated by a randomized controlled design is now necessary. There is a clear need to clarify the clinical effectiveness of specific, yet multicomponent or bundled, nurse-led family interventions [24] that build on guideline-based recommendations for family care in the ICU [15]. Two groups have already tested similar multicomponent interventions combining communication and support with a family navigator role [30, 38], which have shown promising effects on the quality of family care but less clear effects on post-ICU family member health.

The FSI adds, in addition to engagement/liaison and communication, a family systems intervention component. This component, delivered through therapeutic family conversations, is based on a relational family systems nursing approach and includes assessing family structure, processes, and resources, and supporting families through relationship-focused and psycho-educational interventions at the family systems level [52, 53, 55]. Such a systemic family intervention has been found to be effective in chronic illness [54, 123, 124]. In the context of critical illness, one group has pilot-tested a single component family health conversation intervention, delivered in the early post-ICU phase [125]. They found that families who received the intervention were able to improve their family and social functioning from baseline to month three, and demonstrated better functioning and mental health as well as mental health after twelve months. The FSI, while building on existing knowledge, denotes a novel, feasible and acceptable nurse-led family intervention in ICU, adding a relationship-focused, systemic component to the engagement/liaison and communication/shared-decision-making, spanning from ICU admission into the early post-ICU phase [33].

In addition to the well-established service and clinical outcomes that such family interventions seek to improve, we added family management as a third relevant clinical outcome [125]. We also chose to target a more general critically ill patient group to account for those family members whose close others survive critical illness. Critical illness survivors exhibit high levels of post-ICU physical, cognitive, and mental impairments, requiring considerable and often new-onset family caregiving [8, 68, 126–128]. Given the increased prevalence of critically ill patients due to the COVID-19 pandemic, who have a high prevalence of post-intensive care syndrome [129, 130], more families are affected by the health impact of critical illness. In addition, pandemic-related access restrictions increase family suffering and risk for negative health outcomes [12, 13, 19].

Patient and family member representatives are involved in the trial design and implementation [46, 131]. They actively collaborate with the research team in ensuring that participation in the FICUS trial is meaningful and feasible. In addition, they participate in activities of the FICUS study group and meet regularly to advise the lead researchers on study processes. Patient and public involvement (PPI) has been increasingly called for to ensure the relevance, feasibility, and impact of clinical and critical care research [45, 46, 132]. It has been reported that many challenges often inhibit meaningful engagement and effective collaboration [133, 134]. Hence, the early installment and ongoing partnership with the patient and family representatives, together with the integration of the patient liaison in the research team, build a solid foundation for the implementation of effective PPI in the FICUS trial.

In conclusion, the FICUS trial will evaluate a nurse-led, multicomponent, bundled family support intervention that is embedded in interprofessional care delivery in the ICU and aims to generate high-quality evidence on intervention effectiveness and knowledge of its implementability. Both types of evidence are necessary to determine whether the intervention works as intended in clinical practice and could be scaled up to other ICUs. The study findings will make a significant contribution to the current body of knowledge on effective ICU care that promotes family participation and well-being.

### Trial status

At the time of manuscript submission, the FICUS trial has been approved by the responsible Swiss cantonal ethics committees (Nr. 2021-2300). Fifteen of the sixteen required clusters have been recruited. Enrollment of the first participant is expected in spring 2022. Recruitment is expected to be completed by early 2024.

Protocol version: 1.0, 25 October 2021.

## Supplementary Information

**Additional file 1.** SPIRIT Checklist.

## Data Availability

Data sharing is not applicable to this article as no datasets were generated or analyzed during the current study (study protocol).

## Abbreviations

BRS: Brief Resilience Scale
DT: Distress Thermometer
EDC: Electronic Data Capture
FAD-GF: Family Assessment Device - General Functioning Scale
FICUS: Family in Intensive Care UnitS
FSI: Family support intervention
FS-ICU-24R: Family Satisfaction in ICU Questionnaire Revised
eCRF: Electronic case report form
HADS: Hospital Anxiety and Depression Scale
ICC: Intra-cluster correlation coefficient
ICE-FPSQ: Icelandic Family Perceived Support Questionnaire
ICU: Intensive care unit
IES-6: Impact of Events Scale-6
MDSi: Minimal Data Set
MRC: Medical Research Council
QoL-VAS: Adapted VAS on Quality of Life
QQPPI: Questionnaire on the Quality of Physician-Patient Interaction
pCRF: Paper case report form
PFAG: Patient and Family Advisory Group
PICS-F: Post-intensive care syndrome-family
PPI: Patient and Public Involvement
RCT: Randomized controlled trial
SAPS-2: Simplified Acute Physiology Score
SGI: Swiss Society for Intensive Medicine
SWLS-5: Satisfaction with Life Scale
WHO-5: WHO-5 Well-being Index
